# *In-Silico* Trials for Treatment of Acute Ischemic Stroke

**DOI:** 10.3389/fneur.2020.558125

**Published:** 2020-09-16

**Authors:** Praneeta R. Konduri, Henk A. Marquering, Ed E. van Bavel, Alfons Hoekstra, Charles B. L. M. Majoie, The INSIST Investigators

**Affiliations:** ^1^Biomedical Engineering and Physics, Amsterdam University Medical Centers, Amsterdam, Netherlands; ^2^Radiology and Nuclear Medicine, Amsterdam University Medical Centers, Amsterdam, Netherlands; ^3^Computational Science Lab, Institute for Informatics, Faculty of Science, University of Amsterdam, Amsterdam, Netherlands

**Keywords:** INSIST, *in-silico* clinical trials, acute ischemic stroke, *in-silico* modeling, virtual patients, virtual populations, validation, simulation

## Abstract

Despite improved treatment, a large portion of patients with acute ischemic stroke due to a large vessel occlusion have poor functional outcome. Further research exploring novel treatments and better patient selection has therefore been initiated. The feasibility of new treatments and optimized patient selection are commonly tested in extensive and expensive randomized clinical trials. *in-silico* trials, computer-based simulation of randomized clinical trials, have been proposed to aid clinical trials. In this white paper, we present our vision and approach to set up *in-silico* trials focusing on treatment and selection of patients with an acute ischemic stroke. The INSIST project (*IN-Silico* trials for treatment of acute Ischemic STroke, www.insist-h2020.eu) is a collaboration of multiple experts in computational science, cardiovascular biology, biophysics, biomedical engineering, epidemiology, radiology, and neurology. INSIST will generate virtual populations of acute ischemic stroke patients based on anonymized data from the recent stroke trials and registry, and build on the existing and emerging *in-silico* models for acute ischemic stroke, its treatment (thrombolysis and thrombectomy) and the resulting perfusion changes. These models will be used to design a platform for *in-silico* trials that will be validated with existing data and be used to provide a proof of concept of the potential efficacy of this emerging technology. The platform will be used for preliminary evaluation of the potential suitability and safety of medication, new thrombectomy device configurations and methods to select patient subpopulations for better treatment outcome. This could allow generating, exploring and refining relavant hypotheses on potential causal pathways (which may follow from the evidence obtained from clinical trials) and improving clinical trial design. Importantly, the findings of the *in-silico* trials will require validation under the controlled settings of randomized clinical trials.

## Introduction

Endovascular treatment (EVT) has become the standard of care for patients with acute ischemic stroke (AIS) after its benefit was demonstrated by multiple randomized clinical trials (RCTs) (1). Despite improved functional outcome after EVT, up to 66% patients have an unfavorable outcome and remain functionally dependent (1–3). Functional outcome, generally assessed 90 days after stroke onset, predominantly depends on the patient's baseline characteristics including but not limiting to age (4), previous comorbidities (5), stroke severity (4, 6), collateral capacity (7, 8), and thrombus characteristics (9, 10). Delay to receive care strongly reduces the effect of treatment (11–13). Furthermore, ischemic lesion characteristics like volume and location, before and after treatment are also known to be strong predictors of functional outcome after 90 days (14–19).

New AIS trials are focusing on testing new thrombolytics, improved stent designs and testing the applicability of thrombectomy to previously understudied patient sub-groups. However, not more than 10% of the compounds that are tested in clinical trials get launched in the market (20). By design, RCTs do not serve the purpose of explaining the ineffectiveness of treatments. However, this is a task that could be performed with *in-silico* approaches (21). Further analysis to explain the established efficacy of a treatment by *in-silico* methods may allow for generation of potential hypotheses. Before these can be introduced in clinical practice, valiation by RCTs is mandatory. Computational or *in-silico* modeling is playing an increasing role in research and development of biomedical products and is acknowledged as an alternative to animal studies in some preclinical trials by regulators (21–23). Statistical models that accurately describe the most important patient characteristics can generate “virtual patients.” Combining such virtual patient populations with *in-silico* models (ISMs) of disease and treatment will help to set up *in-silico* trials (ISTs). In such ISTs, virtual patients receive virtual treatments and effect of treatment on clinical outcome is estimated (21). This project aims to develop a platform that enables the execution of ISTs for AIS. The proposed IST platform aims to be a proof-of-concept to investigate the extent to which *in-silico* modeling can accurately simulate bench-testing, animal testing, and clinical trial results. After validating the proof-of-concept, some plausible hypotheses may emerge due to the hypotheses-generating nature of this approach. Although ISTs will not allow for testing these hypotheses, they will be useful in optimizing trial design, may provide potential explanation into the causes of (un-) planned effects including less probable clinical situations (21). We envision that developing such a platform can considerably contribute towards a depper understanding of the etiology and pathophysiology of AIS and its treatment effects at the patient and population levels. In the following sections, we describe a quantitative approach to develop a platform that can execute, validate ISTs for AIS, generate and refine hypotheses on the potential successfulness of new treatments, the suitability of treatments for specific patient populations and to provide tools for *in-silico* evaluation of trial design modeling.

## Methods

To develop and validate a platform to execute an IST, we intend to implement a 3 fold approach. We want to generate virtual populations of AIS patients and develop ISMs for (1) thrombosis and thrombolysis, (2) intra-arterial thrombectomy and (3) microvascular perfusion, cell death, and recovery of brain tissue after reperfusion based on anonymized clinical, imaging, and thrombus histopathological data from the Multicenter Randomized Clinical Trial of Endovascular Treatment for Acute Ischemic Stroke in the Netherlands (MR CLEAN) trial, the MR CLEAN Registry and the HERMES collaboration (1, 2, 24). We will validate these ISMs using laboratory experiments and available anonymized clinical data. We aim to apply these ISMs to virtual patient populations with AIS with the goal to generate an IST platform, followed by validation and application of the IST platform.

### Patient Population

Anonymized baseline (clinical and imaging) data, treatment characteristics and outcome (clinical and imaging) data from patients included in the MR CLEAN trial (2). MR CLEAN Registry (24) and the HERMES collaboration (1), totaling over 4,500 patients will be used to develop, execute, and validate the ISMs and ISTs. Anonymized data from on-going RCTs in AIS patients within the Collaboration for New Treatments of Acute Ischemic Stroke (CONTRAST) consortium (www.contrast-consortium.nl) comprising of ~2,500 patients will also be included in this project. The anonymized data from the HERMES (1) and CONTRAST collaboration will be used to validate the ISTs.

### Design

**Figure 1:**
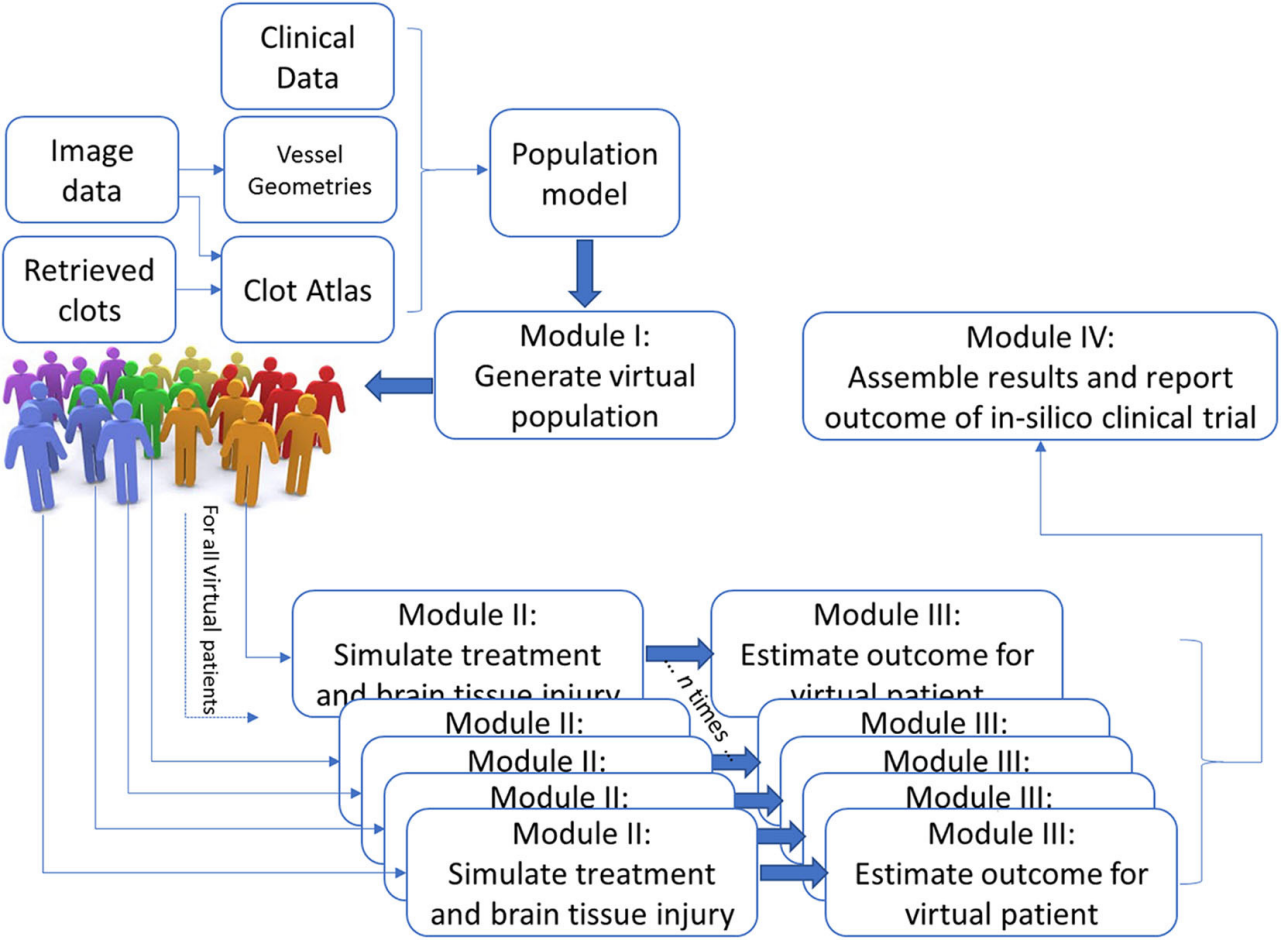
Schematic overview of the INSIST approach to implement an IST for AIS.

The IST consists of four main software modules (Figure 1). The first module generates virtual populations of AIS patients; the second simulates treatment and brain tissue injury; the third estimates outcome for each individual virtual AIS patient and the final module assembles all results and reports the outcome.

#### Module I: Population Model to Generate Virtual Populations of AIS Patients

We aim to generate virtual populations of AIS patients, that are defined using probability density function(s) over all relevant patient specific characteristics, including correlations, and interactions between them, as required by the computational models in Module II and statistical models in Module III. To mitigate the effects of selection bias, the virtual population model is based on anonymized data from the MR CLEAN Registry: a nationwide registry that includes all patients with an AIS due to a large vessel occlusion in the anterior circulation that received endovascular treatment; and not on data from a trial population with very strict inclusion and exclusion criteria.

The virtual population model is developed using 15 characteristics including, clinical (age, sex, systolic blood pressure at admission, pre-interventional modified Rankin Scale, NIH Stroke Scale, comorbidities like previous stroke, diabetes mellitus, atrial fibrillation), workflow based (time from onset to emergency room, time from emergency room to groin puncture), imaging (occlusion location, collateral score, ASPECTS), and clot characteristics (presence of a hyperdense artery sign on baseline NCCT, percentage of fibrin/RBC based on histological analysis). These characteristics have been selected based on prior clinical knowledge of clinicians within the consortium, previously validated stroke prediction platform (25) and requirements of the ISMs of treatment. By mainly deriving the characteristics from a previously validated prediction model and using a relatively large dataset approximating over 4,500 patients, we limit the effects of over-fitting. To improve the accuracy of estimating the correlations of population characteristics (for example—percentage of fibrin/RBC) that are only available for a subset of the dataset, correlations of such characteristics are only estimated with characteristics that are available for the entire population and that they are most associated with to improve accuracy (for example—occlusion location).

Furthermore, the virtual populations are developed using the vine copula model. Compared to state of the art techniques like conditional regression and imputation methods, the vine copula model has several advantages including capturing interactions between the characteristics describing the population as lower levels of dependence structures (26–28).

##### Vessel geometry, clot atlas and ischemic core atlas

Validated (semi-) automated quantitative methods to extract information from radiological images like collateral capacity (29), early ischemic changes (30), thrombus characteristics (10, 31), infarct (32), and hemorrhage (33) characteristics are available to determine radiological characteristics of follow up. We are creating a library of vessel geometries based on the intracranial arteries segmented from baseline imaging data. Segmentation of intracranial arteries is performed using artificial intelligence based techniques. We are also identifying and categorizing different phenotypes of aortic arch-types and intracranial vascular anatomies. In addition, we are performing analysis of multiple thrombus features [e.g., size (length, volume), location, perviousness, etc.] obtained from baseline imaging data (10). We are extending previous methods to create ischemic core atlases to predict the size and location of the final infarct based on the thrombus location, composition, and collateral score. Furthermore, we are creating probability maps of infarcts that indicate the chance of having an infarct in a specific region based on prior clinical, imaging, and treatment information (34).

##### Relationship between clinical, imaging, and histological characteristics of thrombi

We analyze the clots retrieved after EVT procedure for patients within the MR CLEAN Registry (24) using routine macroscopy, histology with various stainings and with immuno-histochemistry using confocal microscopy and (immuno-) scanning electron microscopy. We quantify the thrombus components and their contribution to the thrombus. We also perform electron microscopy of the stents to study the stent-thrombus interaction and its dependence on clot composition (35, 36). With these measurements we will create a clot database and use it to generate a clot population model after incorporating clinical characteristics (such as age, sex, stroke etiology, and prior IV-alteplase), procedural characteristics (time from onset to alteplase, time from alteplase to intra-arterial thrombectomy), and interventional characteristics (stent-retriever, aspiration).

##### Generation and validation of virtual patients and populations

Based on the population models described in the previous sections, virtual patients are created by randomly sampling from the distributions, while accounting for inclusion criteria that may be required in a specific IST. A virtual patient is defined as a combination of characteristics including clinical, anatomical data, and clot specifics, and is used as input to the computational models in Module II. We will combine a large number of individual virtual stroke patients to generate virtual cohorts. Specific virtual cohorts can be generated by setting characteristics, such as age, sex, and baseline NIH Stroke Scale, like setting in- and exclusion criteria in RCTs (26–28).

#### Module II: Simulate Treatment and Brain Tissue Injury

We intend to simulate brain perfusion and growth of an ischemic lesion after AIS (pre- and post-treatment) for every virtual patient generated in Module I. Thus, the output of this Module will include an estimate of the lesion characteristics like location and volume, and status of recanalization and reperfusion for every virtual AIS patient.

**Figure 2:**
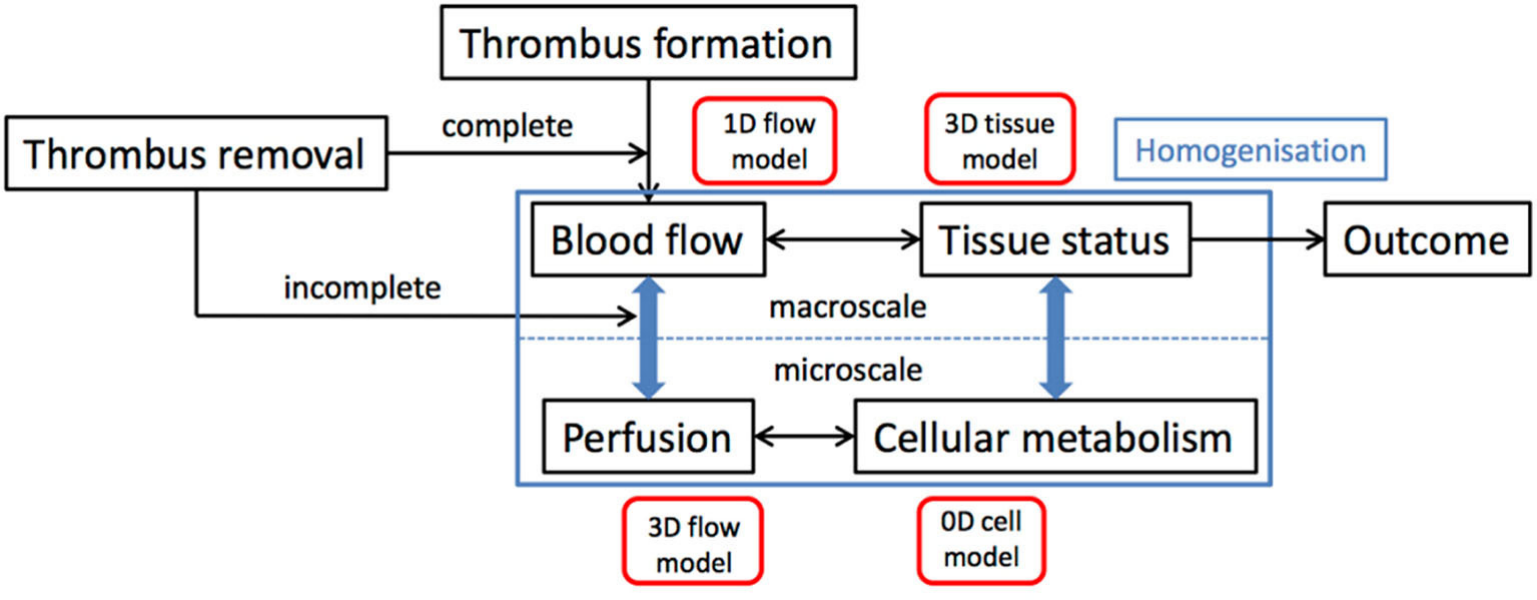
Schematic overview of the multiscale model that simulates AIS and its treatment.

The onset of AIS serves as a starting point of the simulations in this Module. Clot characteristics like location and size and time from onset to treatment are derived from the population model. We then simulate the flow adjustment in response to the occlusion of artery in 1-D models for the large blood vessels that are coupled to simulations of brain tissue perfusion. Treatment, modeled as the (partial) opening of the arterial segment, mimics the details of thrombolysis, and thrombectomy. We also incorporate the possible spray of micro-emboli in the treatment models. The redistribution of the blood flow in the brain is then fed to a 3-D homogenized tissue level perfusion model that describes the (lack of) perfusion of the brain over time, which is the input for the homogenized 3-D brain level stroke model. Figure 2 shows a schematic overview of this multiscale model of AIS and its treatment.

##### *In-silico* models for simulation of thrombosis and thrombolysis

Within INSIST, we develop numerical mathematical models, based on bio-physical principles, in order to explain and predict the processes of thrombosis and thrombolysis relevant to stroke events. The main goal is to understand the formation of the clot, as well as its 3D structure (porosity, composition, inhomogeneity) and what makes some patients resistant to thrombolysis. The proposed models include the most relevant blood factors, nature of treatment, vessel geometry, and flow properties to simulate various patient specific scenarios. The mathematical models include two levels of description: first a 1D macroscopic approach, based on a system of partial differential equations, with validated bio-physical reaction constants, but limited to simple geometries and second, a 3D mesoscopic model able to consider arbitrary flow situations and an explicit structure of the clot. The second model implements the thrombosis and thrombolysis processes as dynamics of idealized particles, transported by a Lattice Boltzmann fluid. These two models typically return lysis time and lysed volume for different situations (depending on flow condition, clot structure, blood composition, etc.). They are being validated with *in-vitro* experiments, performed by INSIST partners.

##### *In-silico* models of mechanical thrombectomy

We are developing ISMs of the stent-retriever, stent placement and stent-thrombus interaction that are driven by *in vitro* and *ex-vivo* experiments with and without thrombolysis treatment. The developed models generate measures of recanalization and clot fragmentation. We are also incorporating the biomechanics of thrombus-vessel adhesion into these models. By simulating the interaction between the stent-retrievers and the vessel wall, we will be able to assess the potential for tissue damage associated with the forces applied by the retriever during deployment and shear stress induced during the retrieving operation. These ISMs are be based on the established techniques of finite element modeling of stent-retrievers (37, 38).

##### Blood flow, perfusion, and microcirculation modeling

With the vessel models, we can generate virtual patient specific 1D blood flow models for the large conductance vessels in the brain (39–43). We are extending these models toward the smaller pial vessels, covering the leptomeningeal collateral network (44, 45). Since there are hundreds of millions of arterial and arteriolar segments in the human brain, modeling individual segments below a certain branching level is neither feasible nor required. We, therefore, use homogenized models for the distribution of number and size of penetrating vessels over the cortex and for the downstream microvascular beds. Such homogenized models are based on porous media physics and include a description of perfusion in terms of Darcy's law, considering the anisotropic nature of the vascular bed (46, 47). Essentially all arteries, but notably the arterioles have significant resistance for perfusion. Control of arteriolar diameter by smooth muscle contractile activity is a core process in autoregulation of brain perfusion, and this process is strongly affected in AIS and during reperfusion (48). The models will be validated in individual subjects through angiographic imaging and perfusion mapping and will be used to assess the robustness of the circulatory pathways to alterations in supply or to changes in individual vessels (for example micro-emboli).

##### *In-silico* ischemia models

We will couple models of the cell response to hypoxia with models of hypo-perfusion injury (49–51). Using homogenization techniques, the models of the microvasculature can be scaled across multiple length scales, which will result in a full advection-convection-diffusion equation, to be performed in the context of both a response to a large clot in a large supply vessel and to micro-emboli in multiple vessels. The resulting 3D model of hypo-perfusion injury will consider the whole cycle of hypo-perfusion, tissue damage and reperfusion within a single, dynamically varying, model for the first time. We will combine models of cerebral blood flow with those of cerebral metabolism, using multi-scale techniques to develop models that incorporate the behavior at both the cellular level and the tissue level.

#### Module III: Outcome Estimation at Patient Level

Characteristics of the final lesion like location and volume are known to be important indicators of patient outcome (15, 19). In this Module, a statistical model that correlates the output of Module II in combination with clinical parameters to patient outcome is developed. Specifically, we will statistically estimate early neurologic deficit as a primary outcome measure. Adverse events, including intracranial hemorrhages, will not be modeled by the *in-silico* models, but will be statistically estimated. Modified Rankin Scale after 90 days is then predicted based on the estimated treatment success, early neurological deficit and adverse events. By executing Modules II and III for each virtual stroke patient in the virtual population, we generate an output dataset that includes the recanalization and reperfusion status, infarct characteristics and functional outcome for each virtual patient.

#### Module IV: Report Outcome at Population Level

In this Module, we will translate and aggregate the results obtained from the previous Module for each individual virtual stroke patients and report results on the population level, such that comparison with real RCTs becomes possible. The Modules mentioned above are implemented as standardized and stand-alone software packages. The overall IST is then implemented as a standard workflow, for which we will be using the Kepler system (see https://kepler-project.org) to enable easy plug-and-play features.

#### Validation

Evaluating the credibility of an IST requires careful and complete validation of all separate components that constitute the trial, and the validation of the complete integrated IST. We will validate the integrated ISMs that are applied to the virtual populations of AIS patients using the anonymized data from the associated trials. We will simulate the trial population and treatment as performed in each of the 7 RCTs, with the IST platform developed in this project using anonymized data from only 6 RCTs (1, 2). We will also perform blinded comparison with the recently started CONTRAST trials (www.contrast-consortium.nl). Specifically, we will compare the distributions and correlations of this virtual cohort to the baseline characteristics of the population enrolled in simulated trial and provide measures of model performance such as calibration and discrimination. Our validation will follow the formal Verification, Validation, and Uncertainty Quantification (VVUQ) procedures as outlined by the ASME V&V40 subcommittee on verification and validation in computational modeling of medical devices, which was published in early 2017 (52) and which FDA has adopted for computational models used for clinical decision support for introduction of new devices.

## Anticipated Results

We believe that accurate tailoring of the above-mentioned Modules will help to build a platform that can be used to execute and conduct ISTs. We have identified three key hypotheses that would be of interest to healthcare professionals, modelers, and pharmaceutical and device industry to explore and refine using the validated IST platform:

- Alternative configurations of stent-retrievers, local distal access catheters, aspiration devices, and balloon guide aspiration devices that can reduce thrombus fragmentation will result in improved treatment outcome.
- Drugs that reduce active Thrombin-Activatable Fibrinolysis Inhibitor (TAFIa) can improve recanalization by tissue plasminogen activator and provide better microvascular reperfusion after thrombectomy.
- Patients with AIS due to large vessel occlusion benefit from an early start of thrombectomy, before the administration of thrombolysis.

We will focus on the optimization of medication administration (pharma industry), use of medical devices (device industry) to improve procedural and peri-procedural aspects of therapy and improved patient stratification for personalized thrombolytic and/or thrombectomy treatment (clinicians).

## Discussion

In this white paper, we describe a methodology that should enable an initial assessment of the added value of ISTs and provide insights into the best practices in setting up such ISTs. We believe that the results of the *in-silico* platform may provide information at a population level given the cohort characteristics, and not on the individual patient level for clinical decision making. Nevertheless, the results of the *in-silico* platform can provide insight on the efficacy of new treatments. The ISTs can be performed for different virtual populations. In all cases, one of our end goals will be to optimize the design of a real-life clinical trial. Hence, we will develop and implement protocols that will enable us to run all ISTs multiple times sweeping over parameters that need to be optimized in a(n) (computationally) efficient way. By the end of the project we will have obtained a much deeper understanding of the pathophysiology of AIS and reasons for failure of current treatments, have delivered and assessed ISMs that explain treatment efficacy. Moreover, the accuracy of ISTs will be assessed by comparing their results with the findings from running and recently completed clinical trials.

We envision that an integrated approach of multiple ISMs in combination with accurate virtual populations of stroke patients will provide valuable insights for the design of relavant trials and thus, contribute to improved biomedical products and treatment success. Nevertheless, ISTs cannot replace clinical trials as ISMs and ISTs are based on data generated from the controlled settings of clinical trials. However, as advertised now by the FDA and EMA, ISTs have value to improve clinical trial design and enhance the level of evidence so that less clinical trials are required before approval from the regulatory bodies.

There are various foreseeable limitations to this study. We must acknowledge that modeling brain tissue infarction, and the complex chain of events that all play a role in brain perfusion and metabolism is very challenging. We still lack basic knowledge of the underlying physiology, as well as sufficiently detailed experimental data that would allow detailed modeling of these processes. However, by reporting outcomes at a population level and by combining a statistically driven component to the modeling chain (as in Module III), the outcome of the IST can become robust to modeling errors of the individual components of an IST. Detailed validation studies will shed light on this for AIS ISTs and for ISTs in general.

## Conclusion

ISTs have the potential to lead to a more effective human clinical trial design, reduce animal testing, lower development costs, and shorten time to market for new medical products. ISTs also allow improved prediction of human risk for new biomedical products. In addition, there is the potential to reuse the developed ISMs for drug repositioning. Through this project, we aim to show the credibility of ISTs and work toward the regulatory acceptance of *in-silico* computational modeling for decision-making and pre-market submissions.

## Data Availability Statement

Publicly available datasets were analyzed in this study. This data can be found here: https://www.insist-h2020.eu.

## Ethics Statement

Ethical review and approval was not required for the study on human participants in accordance with the local legislation and institutional requirements. The patients/participants provided their written informed consent to participate in this study.

## Author Contributions

All authors contributed to the researched the literature and to the first version of the manuscript. The manuscript has been reviewed and edited by all the other INSIST investigators, and the final version accepted by all authors.

## Insist Investigators

Charles Majoie^1^, Ed van Bavel^2^, Henk Marquering^1,2^, Nerea Arrarte-Terreros^1,2^, Praneeta Konduri^1,2^, Sissy Georgakopoulou^2^, Yvo Roos^3^, Alfons Hoekstra^4^, Raymond Padmos^4^, Victor Azizi^4^, Aad van der Lugt^5^, Diederik W.J. Dippel^6^, Hester L. Lingsma^7^, Nikki Boodt^5,6,7^, Noor Samuels^5,6,7^, Stephen Payne^8^, Tamas Jozsa^8^, Wahbi K. El-Bouri^8^, Michael Gilvarry^9^, Ray McCarthy^9^, Sharon Duffy^9^, Behrooz Fereidoonnezhad^10^, Kevin Moerman^10^, Patrick Mc Garry^10^, Senna Staessens^11^, Simon de Meyer^11^, Francesco Migliavacca^12^, Gabriele Dubini^12^, Giulia Luraghi^12^, Jose Felix Rodriguez Matas^12^, Bastien Chopard^13^, Franck Raynaud^13^, Remy Petkantchin^13^, Vanessa Blanc-Guillemaud^14^, Mikhail Panteleev^15,16^, Alexey Shibeko^15^, Karim Zouaoui Boudjeltia^17^.

^1^Department of Radiology and Nuclear Medicine, Amsterdam UMC, location AMC, Amsterdam, the Netherlands; ^2^Biomedical Engineering and Physics, Amsterdam UMC, location AMC, Amsterdam, the Netherlands; ^3^Department of Neurology, Amsterdam UMC, location AMC, Amsterdam, the Netherlands; ^4^Computational Science Lab, Faculty of Science, Institute for Informatics, University of Amsterdam, Amsterdam, the Netherlands; ^5^Department of Radiology & Nuclear Medicine, Erasmus MC University Medical Center, PO Box 2040, 3000 CA Rotterdam, the Netherlands; ^6^Department of Neurology, Erasmus MC University Medical Center, PO Box 2040, 3000 CA Rotterdam, the Netherlands; ^7^Department of Public Health, Erasmus MC University Medical Center, PO Box 2040, 3000 CA Rotterdam, the Netherlands; ^8^Institute of Biomedical Engineering, Department of Engineering Science, University of Oxford, Parks Road, Oxford OX1 3PJ, UK; ^9^Cerenovus, Galway Neuro Technology Center, Galway, Ireland; ^10^College of Engineering and Informatics, National University of Ireland Galway, Ireland; National Center for Biomedical Engineering Science, National University of Ireland Galway, Ireland; ^11^Laboratory for Thrombosis Research, KU Leuven Campus Kulak Kortrijk, Kortrijk, Belgium; ^12^Laboratory of Biological Structure Mechanics, Department of Chemistry, Materials and Chemical Engineering “Giulio Natta”, Politecnico di Milano, Piazza Leonardo da Vinci 32, 20133 Milano, Italy; ^13^Computer Science Department, University of Geneva, CUI, 7 route de Drize, 1227 Carouge, Switzerland; ^14^Institut de Recherches Internationales Servier, Coubevoie Cedex, France; ^15^Center for Theoretical Problems of Physicochemical Pharmacology RAS, Moscow, Russia; ^16^Dmitry Rogachev National Research Center of Pediatric Hematology, Oncology and Immunology, Moscow, Russia; Faculty of Physics, Lomonosov Moscow State University, Moscow, Russia; ^17^Laboratory of Experimental Medicine (ULB 222 Unit), Université Libre de Bruxelles (ULB), CHU de Charleroi, Belgium.

## Abbreviations

INSIST: *IN-Silico* trials for treatment of acute Ischemic STroke
MR CLEAN: Multicenter Randomized Clinical Trial of Endovascular Treatment for Acute Ischemic Stroke in the Netherlands
EVT: Endovascular treatment
AIS: Acute ischemic stroke
RCT: Randomized clinical trials
ISM: *in-silico* model
IST: *in-silico* trial
VVUQ: Verification, Validation, and Uncertainty Quantification
TAFIa: Thrombin-Activatable Fibrinolysis Inhibitor.
